# Towards advanced bioprocess optimization: A multiscale modelling approach

**DOI:** 10.1016/j.csbj.2023.07.003

**Published:** 2023-07-08

**Authors:** Mariana Monteiro, Sarah Fadda, Cleo Kontoravdi

**Affiliations:** Department of Chemical Engineering, Imperial College London, South Kensington Campus, London SW7 2AZ, United Kingdom

**Keywords:** Bioprocess control, CHO cells, Metabolic optimization, Process systems, Digital twin

## Abstract

Mammalian cells produce up to 80 % of the commercially available therapeutic proteins, with Chinese Hamster Ovary (CHO) cells being the primary production host. Manufacturing involves a train of reactors, the last of which is typically run in fed-batch mode, where cells grow and produce the required protein. The feeding strategy is decided a priori, from either past operations or the design of experiments and rarely considers the current state of the process. This work proposes a Model Predictive Control (MPC) formulation based on a hybrid kinetic-stoichiometric reactor model to provide optimal feeding policies in real-time, which is agnostic to the culture, hence transferable across CHO cell culture systems. The benefits of the proposed controller formulation are demonstrated through a comparison between an open-loop simulation and closed-loop optimization, using a digital twin as an emulator of the process.

## Graphical Abstract

**Figure:**
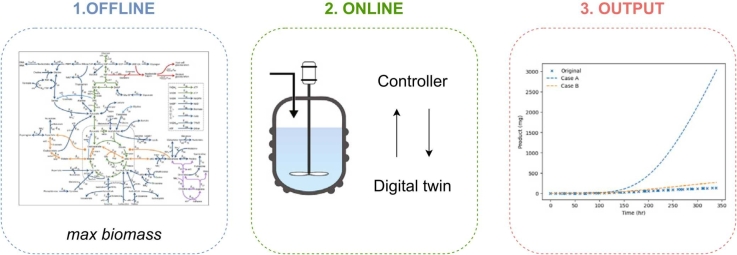

## 1 Introduction

The global biologics market reached an annual value of US$265 billion in 2020, with an expected increase to US$856 billion by 2030 [1]. Mammalian cells produce up to 80 % of the commercially available therapeutic proteins, with CHO cells representing the major fraction of industrial cell hosts [2]. CHO cells produce safe and efficacious protein products, such as monoclonal antibodies (mAbs), which are successfully used, for example, in the treatment of cancer and autoimmune diseases. Despite their commercial success, the production phase of CHO cell-based manufacturing processes is characterized by low product yields (up to 10 *g L* ^−1^ [3]). At these low concentrations, purification becomes the most costly processing step, often accounting for up to 80 % of the total production cost [4]. Several options exist to improve performance, such as optimization of the cell culture conditions (temperature, pH) and feeding strategies. The high experimentation cost and conservative regulatory environment make it challenging to innovate and attempt different feeding conditions without prior knowledge. A strategy to decrease experimentation expenses is through mathematical modelling. Mathematical models have been used to optimize several cell culture operating parameters, such as the medium composition [5], pH [6], and temperature [7] to improve cell growth and antibody production.

Mathematical models can be derived from first principles if they rely on physical, chemical or biological knowledge or data-driven if they solely rely on data. First principles models can be used for prediction; however, the extent of prediction capability greatly depends on the extensiveness of the experimental information on which the model is built. In particular, if data is available for a limited range of process conditions, the model parameter values will likely differ as the operating conditions change. Additionally, the existing data might not capture all the underlying phenomena - a ubiquitous feature in most biological processes [8].

Two key physicochemical phenomena involved in culture processes at the cellular level are transmembrane transport, which governs nutrient uptake and product secretion, and the metabolic reactions in the intracellular environment. Transport phenomena and metabolic reactions are interrelated, so mathematical descriptions of cell culture dynamics should take both into account. Cell culture dynamics can be described at the extracellular level using a Monod-type kinetic model, which, despite being unstructured, can satisfactorily describe cell growth and transport phenomena [9].

At an intracellular level, a kinetics-based approach is intractable as the substantial number of reactions inside the cell would make estimating related kinetic parameters a computationally and experimentally challenging task [10], [11]. An alternative is constraint-based modelling, which formulates the metabolic network as a set of linear equations. If the reaction network is small, then it may be possible to solve via Metabolic Flux Analysis (MFA). However, in most cases, the system is undetermined and therefore needs to be solved as an optimization problem or through flux sampling [12]. The most common method to solve these problems is via Flux Balance Analysis (FBA), which defines the function to optimise based on a cellular objective [13], [14]. Examples include the maximization of growth rate, ATP production, minimization of ATP consumption, or minimization of NADH production [15], [16]. However, a drawback of these methods is that they often underestimate certain important sets of reactions. This could be attributed to the utilization of generic biomass equations or the absence of an energy maintenance constant [17]. Despite this limitation, FBA still provides an upper limit for the maximum cell growth rate and antibody production rate.

Stoichiometric metabolic models provide a static picture of what is happening inside a cell at a given instance. Several authors have solved the models dynamically, optimizing the model for a cellular objective for a time interval. Although the formulation becomes more complex, it allows for the coupling with a model-based control strategy, which requires dynamic integration with time. Several authors have proposed solutions which fit into two major categories: iteratively find a solution for the linear programming problem and integrate the ordinary differential equations for a fixed time until the end of the simulation (Static Optimization Approach) or optimize over the entire solution time (Dynamic Optimization Approach) [15], [18]. The first method has the disadvantage of requiring small time steps to ensure accuracy between iterations. In contrast, the second method turns the problem into a nonlinear programming one, increasing the computational burden considerably. A new method for solving such problems has been proposed, using an interior point approach for the inner linear programming problem [15]. Dynamic FBA has been incorporated into a nonlinear MPC application by coupling the intracellular metabolism with the reactor kinetics for both *Saccharomyces cerevisiae* [19], and *Escherichia coli* cells [20].

While some researchers have used statistical design of experiments to derive transfer functions for control, including pH control using the Plackett–Burman design method [6], others have leveraged mathematical cell culture models to determine the best process inputs to achieve a certain performance offline. Open loop examples include using a Monod-based kinetic model to determine optimal sugar feeding to control antibody glycosylation [21], [22] and using a genome-scale model coupled with a regression model to determine amino acid feeding regimes for the control of three amino acid concentrations [5].

Moving to online control, some studies have proposed simpler control strategies such as glucose setpoint control using Raman spectroscopy for feedback control [23] and online biomass monitoring using a capacitance sensor [24]. There have been model-based closed-loop frameworks proposed in the literature as well. They include controlling the oxygen mass consumed by the cells to steer the process into the target growth rate [25]; coupling in-line Raman spectroscopy monitoring of glucose with a Partial Least Squares model to predict optimal glucose concentration [26]; incorporating and constraining a data-driven model of a perfusion bioreactor to determine optimal temperature, pH, glucose feed concentration and feed and bleed rates [27]. Still in fed-batch antibody production, but with hybridoma cells, authors have also proposed a nonlinear MPC framework, using a Monod-type kinetic model, with an incorporated unscented Kalman filter for state estimation [28].

Although several authors have claimed that MPC has the potential to provide increased product yield, as it has for other industries, it is not widely used either academically or industrially [29]. Advanced control strategies like MPC require models to accurately describe the process in their parametrized conditions [25]. They also require them to be predictive to avoid reparametrization when conditions deviate. Nonetheless, parametrization of cell behaviour is quite a challenging task. The process is influenced by numerous process parameters and is subject to uncertainty stemming from batch-to-batch variability in cell behaviour and challenges in intracellular sample analysis [30].

Model uncertainty and measurement noise are typical problems associated with bioprocesses, directly affecting the generalizability of cell culture models. Besides the technical challenges of implementing MPC, there is also an investment challenge, as online control requires measuring and acting frequently, thus resulting in an infrastructure cost of sensors, actuators and control systems. Lastly, the pharmaceutical industry is heavily regulated, and a change of control system would require approval from regulatory agencies [31].

Table 1 provides an overview of the most significant model-based control strategies in different cell systems. Papathanasiou et al. [32] proposed an *in silico* multi-parametric approach to control the feed flow rate, which is projected to show to lead to higher productivity. Harcum et al. [33] proposed tuning methods of a PID controller for increasing cell growth and productivity of CHO cells. The authors highlighted the importance of improving process modeling as different outcomes can result from the same feeding protocol. Dewasme et al. [28] presented a nonlinear MPC strategy for the control of productivity in hybridoma cell cultures. The authors coupled a Kalman Filter to account for unobserved states of glucose and glutamine concentrations. Nakama et al. [20] proposed a model-based control strategy of *E. coli* based on dynamic FBA. Each of the papers mentioned above showcases some alternatives for controlling cell culture processes. However, certain strategies such as nonlinear MPC [28] and dynamic FBA [20] may be computationally expensive in mammalian systems, while others require online measurements for such short control intervals [32].

**Table 1:** Examples of published work on control strategies in cell systems.

Study	Methodology	Cells
[32]	Multi-parametric MPC for feeding strategies	GS-NSO
[33]	Development of PID controller for glucose and phenylalanine tracking	CHO
[28]	Nonlinear MPC of feeding strategy	Hybridoma
[20]	Optimisation-based control strategies based on dFBA	*Escherichia coli*

In the present work, we propose a MPC strategy that would not require additional investment costs and could be integrated within an already established fed-batch process. Our control strategy circumvents the need for frequent online measurements by considering long control intervals when compared to conventional MPC approaches, presented in the literature [28], [32]. The controller implements optimal feeding strategies by leveraging a reduced-scale metabolic model. The metabolic model provides optimal fluxes of key metabolites (such as glucose, lactate and glutamine) to the controller for five different cell stages. Through optimization, the controller calculates the optimal feeding strategy that leads to the target optimal fluxes. This formulation bypasses the need to solve the metabolic problem dynamically, which, although feasible for other cell types [20], is computationally demanding for mammalian cell systems. The feeding strategy is tested using a digital twin as a surrogate experimental system. We show that optimal feeding strategies lead to higher antibody production in several case studies.

## 2 Materials and methods

**Fig. 1:**
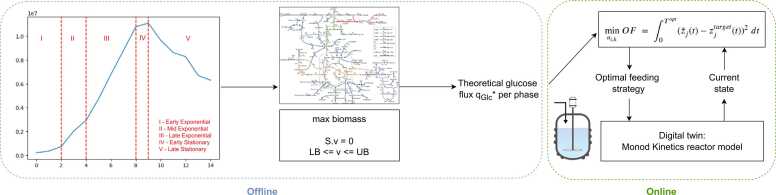
Workflow Diagram. In the offline box, *S* is the stoichiometric matrix, *v* the fluxes of all reactions, *v*_*irrev*_ the fluxes of the irreversible reactions, *LB*_*i*_ the lower bound of flux *i* and *UB*_*i*_ the upper bound. In the online box, *OF* is the objective function, ${\overset{¯}{z}}_{j}\left(t\right)$ the trajectory of the optimization variable *j*, $z_{j}^{target}\left(t\right)$ the target value for the optimization variable, both over time *t*, *u*_*i*,*k*_ the manipulated variable *i* over the control interval *k*, *t* time and *T*_*opt*_ the optimization time horizon.

Fig. 1 represents the proposed control framework. It is comprised of two main sections, offline and online. The theoretical optimum glucose flux through FBA is calculated offline for each cell culture stage. Online, the controller minimizes the difference between current glucose flux versus the online one. The optimal feeding strategy is implemented on a digital twin of the reactor model.

**Fig. 2:**
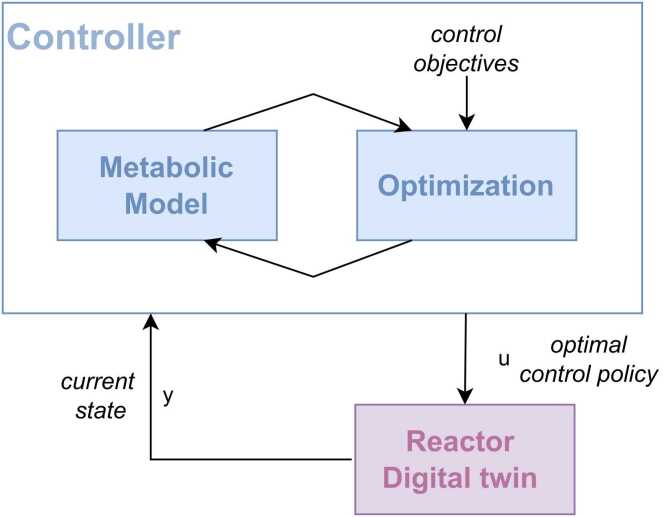
Control framework.

The controller (described in Fig. 1 inside the “Online” box) is depicted in Fig. 2 and can be summarized by following steps: .

1. Get current state *y* (Reactor Digital Twin)
2. Using the current state and the reactor model, compute the optimal control policy (Controller)
3. Implement optimal control policy for control interval 1 in the digital twin of the reactor (Reactor Digital Twin)
4. Repeat steps 1–3 until the end of the simulation

Both the metabolic model and the control optimization were performed using gPROMS ModelBuilder v.7.1.1 (Process Systems Enterprise Ltd). Each component of the control framework will be discussed in the following sections.

### 2.1 Digital twin: reactor model

The reactor model consists of differential and algebraic equations describing the mass balances for each component (cells, nutrients and metabolites) and the corresponding specific uptake and production rates. The cell culture dynamics is described by Monod-type kinetics. The model includes a description of 22 metabolites and an estimation of 35 model parameters. We evaluated the prediction capability of this new model structure using the experimental set-up presented in Kyriakopoulos and Kontoravdi [34] (the cell line used is CHO GS46, and the expressed protein is a chimeric IgG4 monoclonal antibody). We informed the model using two sets of experimental data for the same cell line and medium supplemented with different feed formulations. Fixing the parameters at their estimated values, we compared the simulation results to an independent dataset generated with the same cell line but supplemented with a third feed formulation. The model development and corresponding parametrization strategies can be found in the Appendix (see section A). In Appendix A.3, we also show that the proposed model can accurately predict key performance indicators without needing parameter re-estimation for the new experimental conditions.

### 2.2 Controller

The control strategy is composed of two main optimization steps. The first is to find the glucose flux that leads to optimal cell growth. The second is to determine the optimal feeding strategy that leads closer to that optimal cell growth. The metabolic model is described in section 2.2.1 and the controller optimization is described in section 2.2.2.

#### 2.2.1 Metabolic model

The metabolic model uses a network of 101 metabolites, and 144 reactions [35]. The optimization is formulated as follows:

(1) $$maxv_{biomass}$$

subject to:

$$\begin{array}{c} S.v=0\\ 0<v_{irrev}\\ LB_{i}\leq v_{i}\leq UB_{i} \end{array}$$

where *S* is the stoichiometric matrix, *v* the fluxes of all reactions, *v*_*irrev*_ the fluxes of the irreversible reactions, *LB*_*i*_ the lower bound of flux *i* and *UB*_*i*_ the upper bound. This optimization was performed for each of the five fed-batch stages: early exponential, mid-exponential, late exponential, early stationary and late stationary. The difference between the five stages is expressed in the bounds of the fluxes, which constrain the space to the limits expected for that particular cellular stage. As a result, there will be a set of constant optimal fluxes per culture stage.

In the original paper of the metabolic network, the maximization of biomass is shown to achieve worse predictions than an alternative multi-optimization strategy [35]. The reasoning behind choosing the maximization of biomass strategy is that it is a theoretical upper limit of biomass growth whilst still complying with the stoichiometry of the metabolic model. Using the metabolic model produces an optimal target while still being a realistic objective.

#### 2.2.2 Control optimization

The controller optimization is cast as a minimization between the current and optimal glucose flux computed by the metabolic model (see section 2.2.1).

(2) $$\underset{u_{i,k}}{\min}OF=\int_{0}^{T^{opt}}{\left({\overset{¯}{z}}_{j}\left(t\right)-z_{j}^{target}\left(t\right)\right)}^{2}dt$$

where *OF* is the objective function, *u*_*i*,*k*_ the manipulated variable *i* over the control interval *k*, *t* time, *T*_*opt*_ the optimization time horizon, ${\overset{¯}{z}}_{j}\left(t\right)$ the trajectory of the optimization variable *j* and $z_{j}^{target}\left(t\right)$ the target value for the optimization variable. The following table 2 summarizes the parameter values used in the optimization. The optimization variables have a defined target value. The optimizer uses the manipulated variables to reach a target objective. The control intervals are the fixed time intervals, and they emulate the frequency of measurements. The optimization horizon is the total time considered by the optimizer, which matches the cellular cycles defined experimentally [35]. It is important to note that glucose flux refers to the optimal flux calculated in the metabolic model through biomass maximization. In contrast, the total amount of glucose fed (the glucose feed concentration multiplied by the volume) refers to the optimal feeding strategy determined through controller optimization.

**Table 2:** Optimization Parameters. The extracellular metabolites include glucose, lactate, ammonia, pyruvate, alanine, arginine, asparagine, aspartic acid, glutamine, glutamic acid, glycine, histidine, isoleucine, leucine, lysine, methionine, phenylalanine, proline, serine, threonine, tryptophan, tyrosine and valine.

	Optimization Variables	Manipulated Variables	Control intervals (h)	Control horizon (h)
Case A	All extracellular metabolite fluxes	Glucose Feed Concentration		
		Volume Feed In	8,24,72	72
		Volume Feed Out		
Case B	Glucose flux	Glucose Feed Concentration		
		Volume Feed In	8,24,72	72
		Volume Feed Out		

This study evaluated two optimization cases. All extracellular metabolic fluxes were treated as optimization variables in Case A. On the other hand, Case B focused solely on the glucose flux as the optimization variable. The manipulated variables in both cases were the glucose feed concentration and the volume of feed entering and exiting the system. Although typical advanced control strategies use lower control intervals [36]; we deliberately chose control intervals of 8, 24 and 48 h to replicate the lower measurement frequencies commonly observed in cell cultures. The control horizon is chosen to match each culture phase defined (consult the original paper of the metabolic network for more details [35]). The glucose concentration feed was allowed to vary between 0 and 1000 *mmol*∕*L*_*b*_. *r*. , while the inlet feed was between 0.001 and 1*L*∕*min* and the outlet feed between 0 and 0.05*L*∕*min*. The manipulated variables were defined as piecewise linear.

## 3 Results & discussion

### 3.1 Metabolic model

**Fig. 3:**
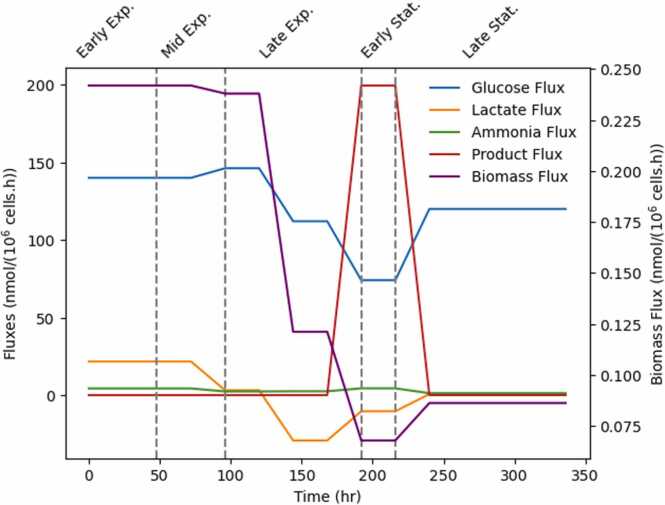
Fluxes of key metabolites (biomass, glucose, lactate, ammonia and product). Grey dashed lines distinguish the different cell culture phases.

Fig. 3 shows the results of the optimal rate of cell growth and the uptake rate of key metabolites as a function of time in days. Growth (biomass flux) is at maximum at the early stages of the batch, decreasing in later stages, being the lowest in the stationary phase when antibody (product flux) is the highest during the early stationary phase. Lactate uptake rates match the lactate shift from production to consumption observed in the experimental data [34]. The predicted uptake fluxes match the predicted ones from the paper that proposed the metabolic network model used herein [35]. The predicted fluxes portrayed in Fig. 3 will be used as control targets for the controller described in the following section 3.2.

### 3.2 Optimal control inputs

**Fig. 4:**
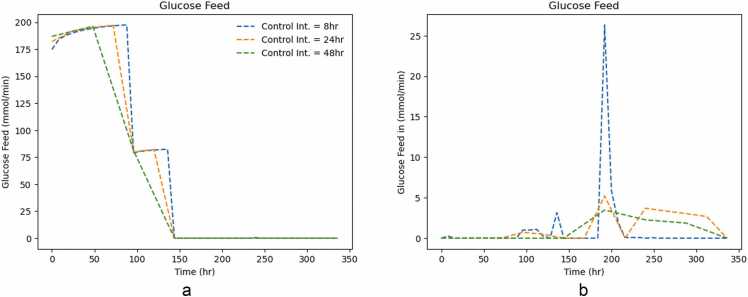
Glucose Feed for both cases for different control intervals. Case A considers all extracellular metabolic fluxes as optimization variables, whereas case B only considers the glucose flux.

Optimal control inputs were computed for different control intervals. Fig. 4(a) and 4(b) show the glucose feed for different control intervals for cases A and B, respectively:

In Case A (Fig. 4(a)), different control intervals display the same trends, except, the shorter the control interval, the longer the feed duration in the first 150 hours of culture. There are three main stages for glucose feed, starting with glucose being fed at 200 mmol/min, and then decreasing to 75 mmol/min. The shorter the control interval, the later does this transitions occurs. The same is observed on the next transition to the 0 mmol/min stage. In all three cases, the majority of the feed occurs during the exponential phase, and decreasing as the batch progresses.

The same is not observed in Case B (Fig. 4(b)), where the feeding trajectory is not step-wise, but rather peak-wise. Longer control intervals lead to shorter but longer (in time) peaks. This is quite noticeable in the shorter control interval case, where the glucose feed peaks from 0 to 25 mmol/min for a short time. In optimal control theory, this might be referred to as ’bang-bang’ control, where the control variables jump from one boundary to another suddenly [37]. An additional difference from case A is that in this case, the glucose feeding seems to be delayed until later in the batch, only starting after two days of the culture.

While shorter control intervals are expected to depict the behaviour of the cell culture process better and, consequently, more accurate control responses, longer control intervals may be better at dealing with measurement noise [38], [39]. This is compatible with the fact that the shorter control interval in Case A depicts an intermediate stage that longer intervals do not. Furthermore, it is compatible with the oscillatory behaviour observed in Case B, for the shorter control interval, as the noise may be causing the observed spikes. A rule of thumb is for the control interval to be 10–20 % of the control horizon. The controller might be pushed to act faster when both the control interval and the prediction horizon are set to 48 h.

The lower sampling frequency might lead to more erratic (and possibly sub-optimal) control decisions. Additionally, the formulation of the optimization problem might be the root cause for such different feeding strategies. In Case A, the optimizer is more restricted than in Case B, given that it has to minimize the trajectory of 22 metabolites (as opposed to just one, glucose). This added flexibility to the controller makes it more susceptible to the control interval.

**Fig. 5:**
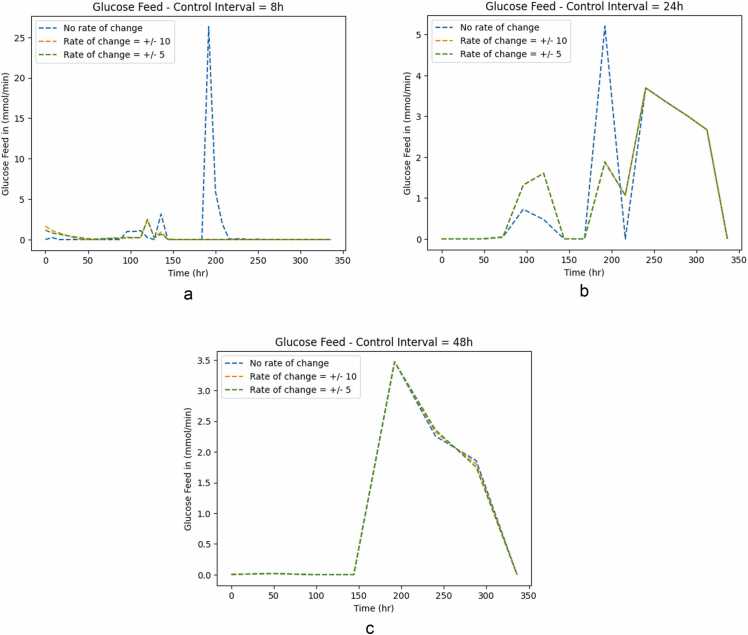
Control input trajectories for case B under different control intervals and different rate of change penalties.

To mitigate the sudden jumps in control inputs, a penalty to the rate of change was added to optimization case B. Two different rate of change penalties were tested: + ∕ − 10 and + ∕ − 5 mmol/L (the same units as the concentration of glucose feed. The rate of change penalties were applied to only one of the three manipulated variables (the glucose feed concentration) as that variable was the one pushing for the sudden jumps. Fig. 5 presents three plots, one for each of the studied control intervals.

The shorter the control interval, the larger was the effect of the rate of change penalty. This is agreement with the fact that the longer the control interval, the smaller the peaks of the control inputs. The results show that the addition of the rate of change is a useful tool to mitigate sudden changes of control inputs, in case they are difficult to implement during an experiment.

**Fig. 6:**
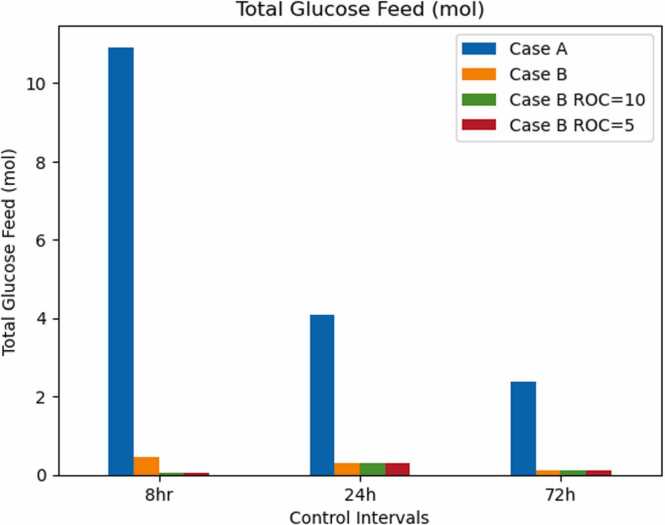
Total Glucose Feed for different control intervals in both optimization cases.

Another aspect to consider is the actual amount of glucose that is being predicted to be fed in both cases. The rate of change penalty was considered as well. Fig. 6 shows the total amount of glucose fed during the cell culture for both cases (in case A, the controller takes into account all extracellular metabolite fluxes in the optimization, whereas in case B the controller only minimizes the difference between glucose flux) and the three control intervals, 8, 24 and 72 h. When accounting for only the glucose flux in the minimization, the optimizer feeds less glucose (Case B) compared to the alternative Case A. This indicates that when the optimizer is forced to satisfy all the metabolic fluxes, it predicts a higher glucose requirement. When the cell’s energy requirements are underestimated, metabolic network models might underpredict the required glucose to be fed. The metabolic model was shown to underpredict growth rate; therefore, it is expected to underpredict the amount of glucose required [35]. That underprediction is expected to be larger as the size of the glucose feed increases, given the lower sampling frequency. This is compatible with the decrease of total glucose feed in both cases. Additionally, the addition of the rate of change penalty came with the cost of an even lower glucose being fed across cell culture duration, which is likely to lead to lower antibody being produced. Hence, the addition of rate of change penalty, whilst useful to mitigate sudden changes in control inputs, might limit antibody production in practice.

**Fig. 7:**
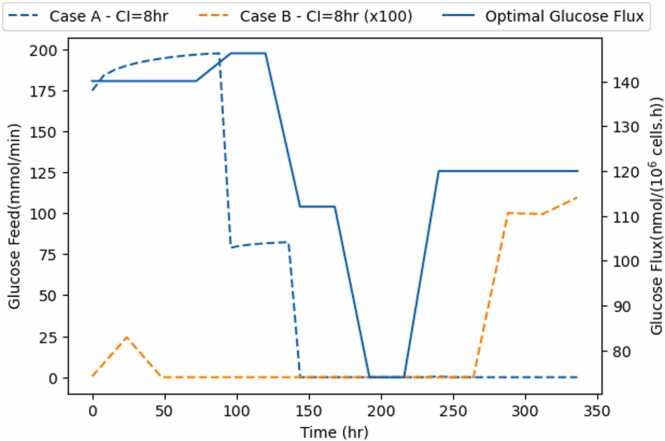
Optimal glucose flux versus optimal glucose feed for a control interval of 8 h, for both cases A and B. Case B has been multiplied by a factor of 100.

Fig. 7 compares the glucose feeding strategies with the optimal glucose uptake rate computed by the network model, for both Cases A and B, for a fixed control interval of 8 h. Case A follows the same pattern as the optimal uptake rate, with a slight advancement. Even though in Case B the optimizer is solely concerned with satisfying glucose flux, the glucose-feeding strategy is less in tune with the glucose uptake rate.

### 3.3 Closed loop results

**Fig. 8:**
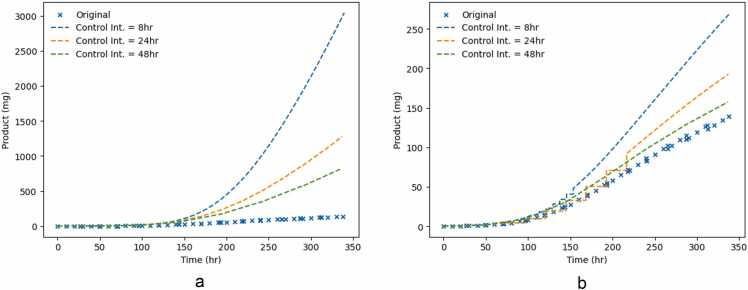
Closed Loop trajectories for total antibody mass produced (in mg) for Cases A and B for different control intervals. Original values refer to the simulation of one feeding strategy described in the digital twin (see Appendix A).

Fig. 8(a) and 8(b) show the closed loop trajectories of both cases for the considered control intervals. The cases are compared against the emulation of one of the experiments used for parameter estimation. The feeding strategies computed by the controller lead to higher antibody production, in both mass and concentration. Case A shows an increase between 83 % and 95 %, whereas Case B between 11 % and 48 %, at the end of the batch. Although in Case A we observe an increase in product quantity, the volume fed is higher, and therefore, the product concentration at harvest is expected to be lower that in Case B.

In both cases, shorter control intervals lead to higher antibody yield. This result is in agreement with other authors: Papathanasiou et al. [32] also found that shorter control intervals lead to higher product quantity, and Sarna et al. [40] reported that shorter horizons lead to a higher quantity of final product in both a constrained and unconstrained control scenario. This agrees with the fact that more frequent samples improve the accuracy of the optimizer. This observed difference is thought to be due to the nature of the optimization problem. The optimizer is far less restricted in Case B. Consequently, the sampling restriction of the different control intervals has an effect. However, in Case A, the optimizer has 23 flux trajectories to minimise, which restricts the solution more than the control intervals.

**Fig. 9:**
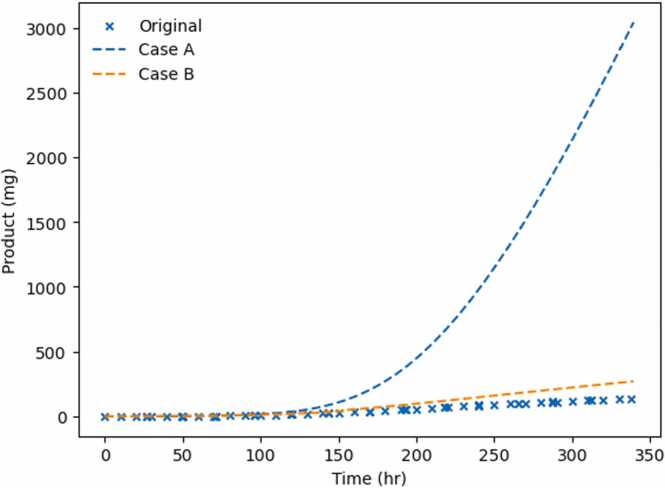
Closed Loop results for Cases A and B, fixing the control interval as 8 h.

Fig. 9 shows the closed loop simulation results for Cases A and B for a control interval of 8 h. Contrary to what might be expected, Case A performs better than Case B. Although the controller is granted higher flexibility in Case B, it performs worse. One of the reasons for this is that in Case B, the total amount of glucose is lower (see Fig. 6). As discussed previously, the total glucose fed is lower due to an underprediction of cell energy requirements.

The closed loop results are compared against an experiment that used a designed feed that had already increased antibody yield up to 3.5-fold compared to batch culture [34]. The results presented in this paper suggest that using an advanced control strategy may lead to even higher antibody production. The proposed method can be used as a more straightforward step towards advanced process control as it does not require online sensors and actuators. It also bypasses the need to develop a complete dynamic metabolic model for CHO cells, which would be computationally challenging.

### 3.4 Controller performance

**Fig. 10:**
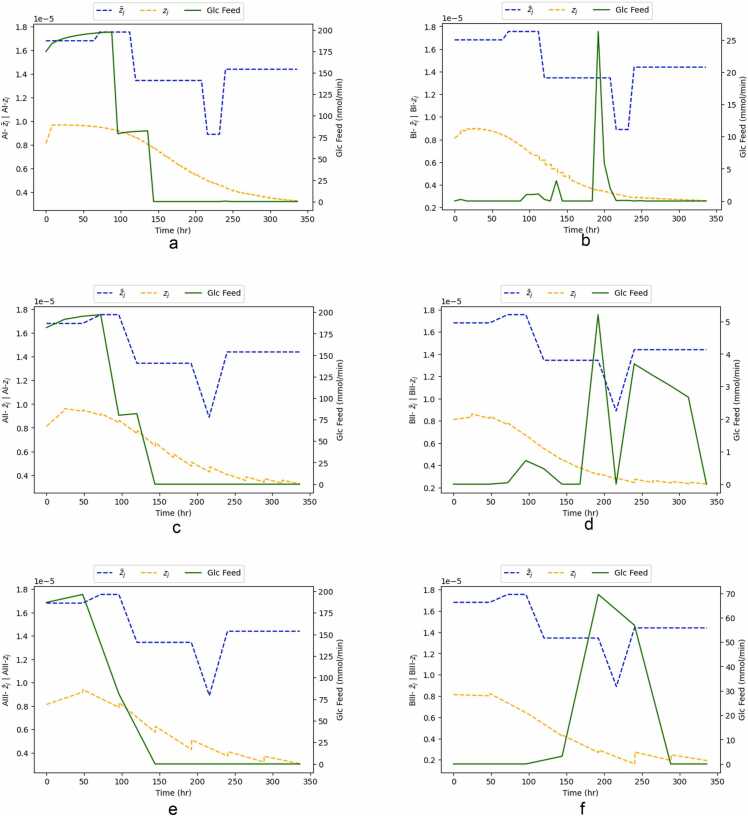
Time course comparison between target glucose flux ($z_{j}^{target}$), optimized glucose flux (${\overset{¯}{z}}_{j}$) and glucose feed, for the two optimization scenarios and the three considered control intervals.

Fig. 10 presents the time course comparison between target glucose flux ($z_{j}^{target}$), optimized glucose flux (${\overset{¯}{z}}_{j}$) and glucose fed for the two optimization scenarios and the three considered control intervals. The target glucose flux ($z_{j}^{target}$) remains the same for cases A and B, corresponding to the values defined in the metabolic model (please refer to section 2.2.1.).

In all case A examples, the glucose feed rises slightly for the first 50–100 h, decreasing for the next 50–100 h and stabilizing at its lowest level in the later stages of the batch. The shorter the control interval, the more piecewise the trajectory is.

Unlike case A, in case B, there is no piecewise trajectory. Glucose feed remains constant in a low value for the first 50–100 h, increasing in peaks as the batch progresses. As discussed in section 3.2, this behaviour might be due to the nature of the controller optimization. In case A, the controller considers the trajectories of 23 metabolites, including glucose. Whereas in case B, the control is relatively less constrained.

It is evident that in both cases A and B, the target glucose flux ($z_{j}^{target}$) and the optimized glucose flux (${\overset{¯}{z}}_{j}$) do not match. In Case A, they are initially aligned during the first 100 h, but this alignment is no longer observed thereafter. However, the target glucose flux $z_{j}^{target}$ and the optimized glucose flux ${\overset{¯}{z}}_{j}$ follow a pattern of overall decrease as the batch progresses. This misalignment between the two might be due to the fact that the target glucose flux, derived by optimizing a metabolic model using FBA, constituting an ideal but practically unattainable scenario.

**Fig. 11:**
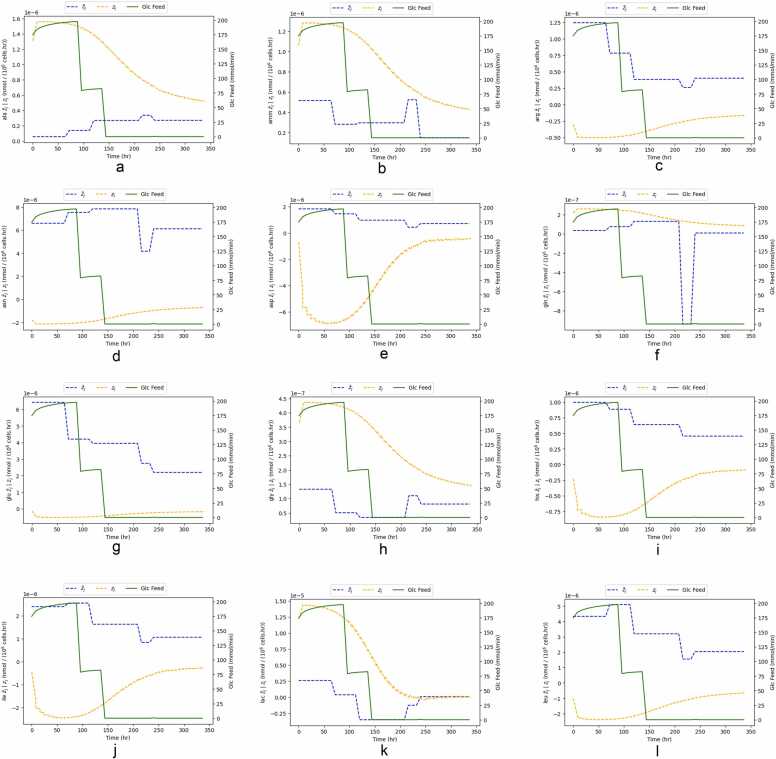
Time course comparison between target metabolite flux ($z_{j}^{target}$), optimized metabolite flux (${\overset{¯}{z}}_{j}$) and glucose feed, for optimization case A and for control intervals of 8 h.

**Fig. 12:**
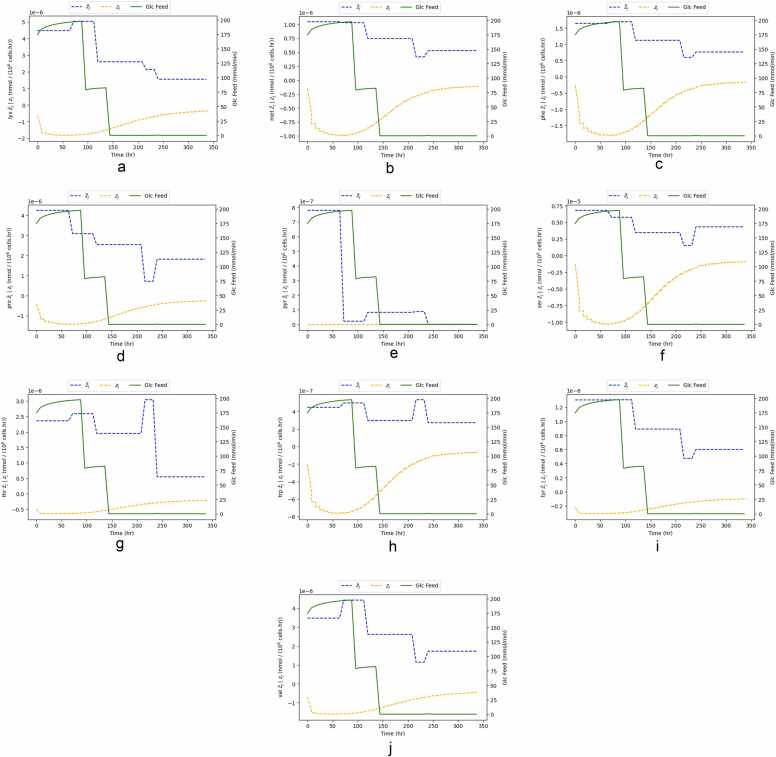
Time course comparison between target metabolite flux ($z_{j}^{target}$), optimized metabolite flux (${\overset{¯}{z}}_{j}$) and glucose feed, for optimization case A and for control intervals of 8 h.

To further understand why case A performs better, we will look into the other 22 metabolites, which are optimization variables for that scenario. Figs. 11 and 12 present the time course of the remaining 22 optimization variables for case A for a control interval of 8 h (the best-performing control interval).

Similarly to Fig. 10, there is a discrepancy between optimized and target fluxes of metabolites. Having 23 optimization variables makes drawing a relationship between control actions and targets more challenging. In all 22 metabolites, the optimized flux gets closer to the target flux as the batch progresses. However, the dynamics of the optimized flux are slower than the target ones. This suggests that the manipulated variables do not have enough leverage to steer the optimization variables into the target values.

**Fig. 13:**
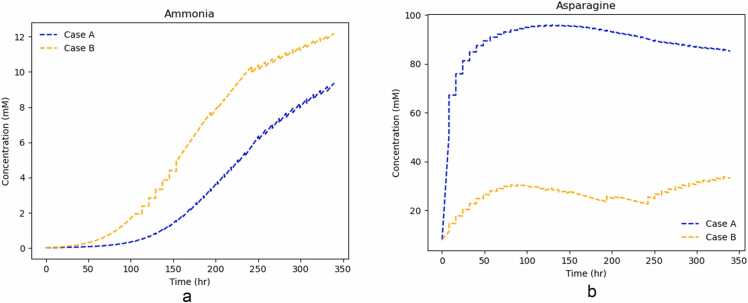
Closed Loop trajectories of extracellular concentrations of metabolites ammonia and asparagine for Cases A and B.

Ammonia and asparagine (Figs. 11d and 11b) are two significant metabolites. Ammonia is considered to directly contribute to cell death (refer to Eq. 15 in the Appendix A). On the other hand, asparagine has been considered a limiting nutrient, whose depletion may lead to cause cell death [41]. The controller pushes ammonia to a lower flux and to an abundance of asparagine. This is confirmed when looking at the time course concentration of these two metabolites. Fig. 13 depicts their closed-loop trajectories of these two metabolites for cases A and B. It suggests that there is better nutrient utilization in case A, given that ammonia is in much lower quantities and asparagine in higher ones.

The optimizations were performed using the nonlinear programming sequential quadratic programming (NLPSQP) solver built into gPROMS ModelBuilder v7.1.1 and run on an Intel(R) Core(TM) i7–10810 U CPU @ 1.10 GHz 1.61 GHz, and each optimization took less than 10 s. There is no significant difference between optimization case A and B. As such, both are computationally inexpensive and suited for online control.

## 4 Conclusions

Herein, we presented a model predictive controller for the production reactor of CHO cell cultures. The control strategy leverages a reduced metabolic network model of a CHO cell to calculate glucose and amino acid uptake rates that will lead to optimal cell growth for each cell phase. These fluxes are used as control targets to compute the optimal feeding strategy. The optimal feeding strategy is then used in a digital twin of the production reactor. Our results demonstrated that this control strategy has the potential to lead to higher antibody production for different cases. Furthermore, our findings support the existing literature by emphasizing the advantages of using shorter control intervals. This suggests that utilizing shorter sampling windows can lead to better controller performance and, consequently, higher productivity. The control framework is flexible and transferable across different CHO cell culture systems. Furthermore, it can include more complex metabolic models, such as genome-scale models. We believe this strategy is an appropriate first step towards advanced process control in the bioprocessing industry as it does not require extensive computational power and can be implemented without extra infrastructure costs.

## CRediT authorship contribution statement

**Mariana Monteiro:** Conceptualization, Formal analysis, Investigation, Methodology, Writing – original draft. **Sarah Fadda:** Formal analysis, Investigation. **Cleo Kontoravdi:** Conceptualization, Funding acquisition, Supervision, Writing – review & editing.

## Declaration of Competing Interest

The authors have no conflict of interest to declare.

## Figures and Tables

**Fig. 14:**
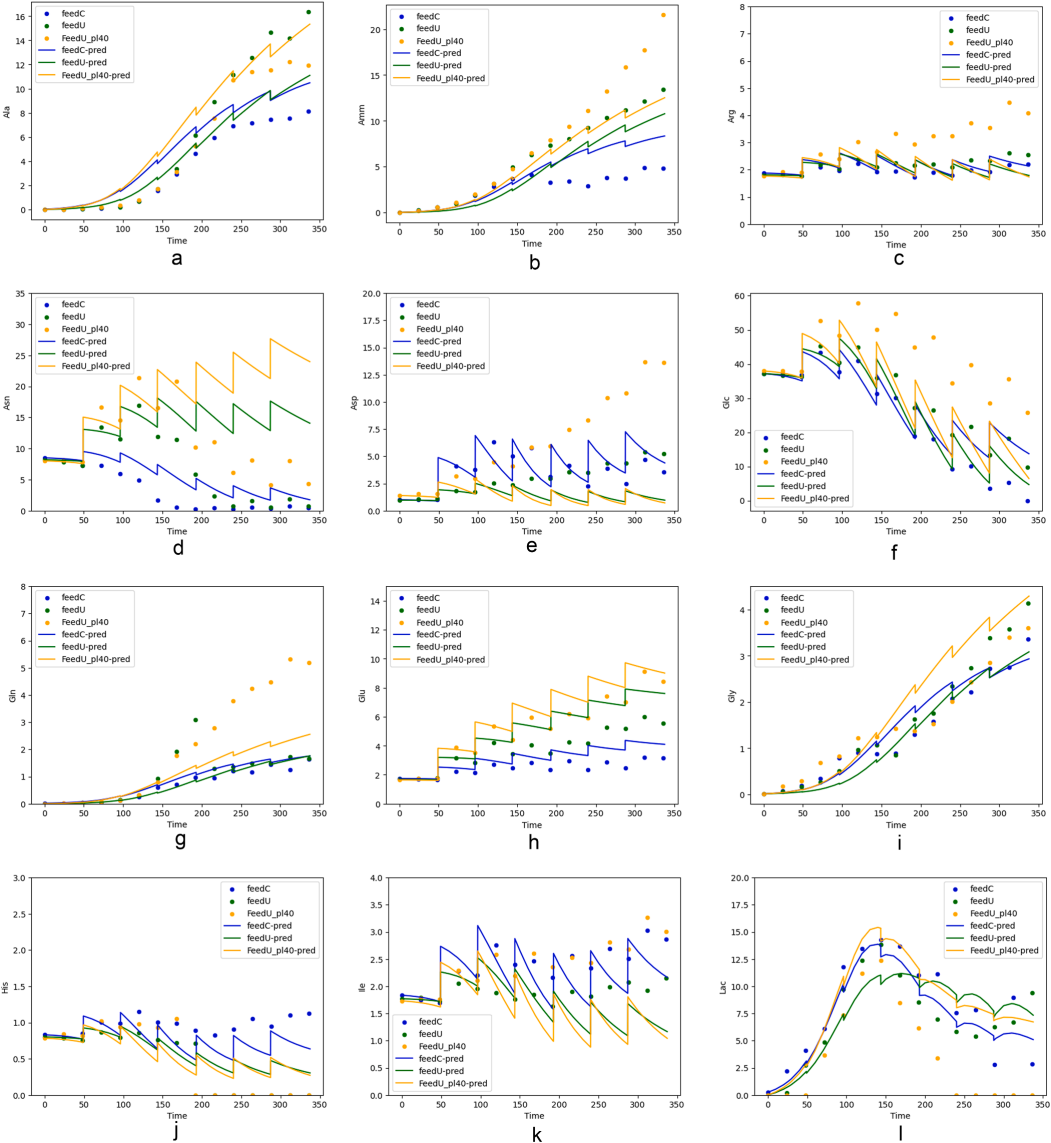
Experimental and simulated concentration profiles of the main nutrients in a culture system fed with feed F_C and F_all_pl40. Experimental and predicted concentration profiles of the main nutrients in a culture system fed with F_all.

**Fig. 15:**
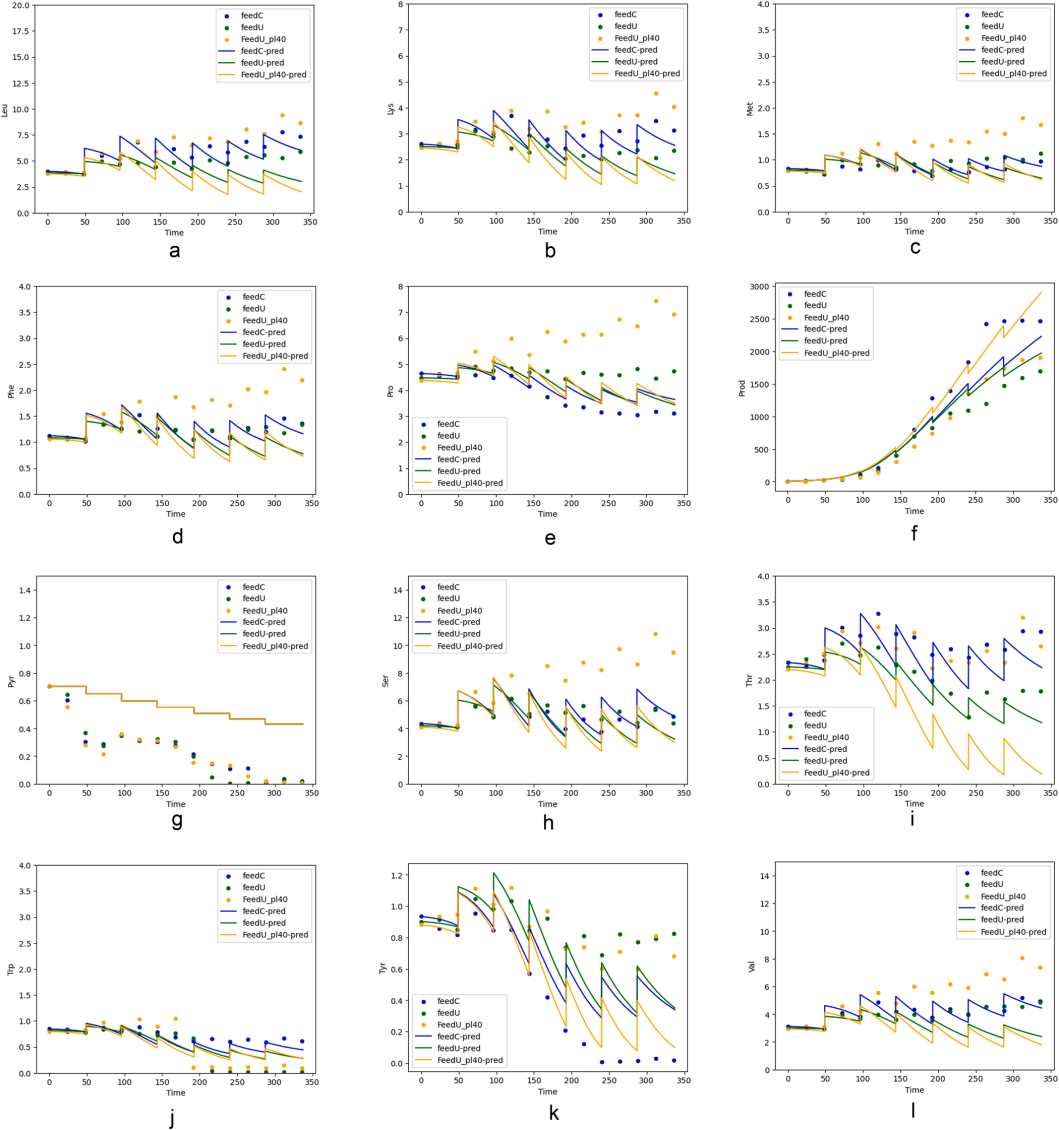
Experimental and simulated concentration profiles of the main nutrients in a culture system fed with feed F_C and F_all_pl40. Experimental and predicted concentration profiles of the main nutrients in a culture system fed with F_all.

**Fig. 16:**
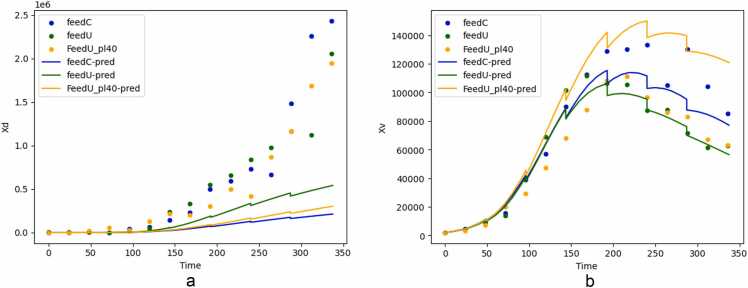
Experimental and simulated cell growth and death profiles in a culture system fed with feed F_C and F_all_pl40. Experimental and predicted cell growth and death profiles in a culture system fed with F_all.

**Table 3:** Fitted model parameters.

Parameter	Unit	Value	Parameter	Unit	Value
*K*_*prod*__,__*t yr*_	mmol/L	0.0165239	*Y*_*Lac*__*/*__*Glc*_	mmol / mmol	1.49530
$k_{d}^{max}$	*h*^−1^	0.0154520	*Y*_*Prod*__*/G*_	mmol / mmol	2.44818x10^6^
*K*_*d*,__*Amm*_	mmol/L	1.0	*Y*_*Prod /NG*_	mmol / mmol	11.0616
*k*_*lac*_	mmol/L	299.384	$Y_{Ala∕X_{V}}$	mmol / mmol	27,582.5
*KI*_*amm*_	mmol/L	4.56167	$Y_{Amm∕X_{V}}$	mmol / mmol	0.600238
*Km*_,__*arg*_	mmol/L	0.996291	$Y_{Arg∕X_{V}}$	mmol / mmol	77,216.9
*Km*_,__*asn*_	mmol/L	0.124390	$Y_{Asn∕X_{V}}$	mmol / mmol	20,146.7
*Km*_,__*asp*_	mmol/L	34.4826	$Y_{Asp∕X_{V}}$	mmol / mmol	1996.86
*Km*_,__*glc*_	mmol/L	1E-6	$Y_{Glc∕X_{V}}$	mmol / mmol	4447.05
*Km*_,__*glu*_	mmol/L	5.0	$Y_{Gln∕X_{V}}$	mmol / mmol	165,551
*Km*_,__*his*_	mmol/L	5.93845	$Y_{Glu∕X_{V}}$	mmol / mmol	68,445.8
*Km*_,__*ile*_	mmol/L	24.3791	$Y_{Gly∕X_{V}}$	mmol / mmol	98,733.5
*Km*_,__*lac*_	mmol/L	45.5977	$Y_{His∕X_{V}}$	mmol / mmol	18,258.7
*Km*_,__*leu*_	mmol/L	5.74809	$Y_{Ile∕X_{V}}$	mmol / mmol	5263.46
*Km*_,__*lys*_	mmol/L	6.73246	$Y_{Leu∕X_{V}}$	mmol / mmol	14,339.6
*Km*_,__*met*_	mmol/L	62.7282	$Y_{Lys∕X_{V}}$	mmol / mmol	15,407.2
*Km*_,__*phe*_	mmol/L	85.8498	$Y_{Met∕X_{V}}$	mmol / mmol	2894.14
*Km*_,__*pro*_	mmol/L	480.846	$Y_{Phe∕X_{V}}$	mmol / mmol	1714.76
*Km*_,__*pyr*_	mmol/L	50.0	$Y_{Prod∕X_{V}}$	mmol / mmol	776.433
*Km*_,__*ser*_	mmol/L	120.487	$Y_{Pyr∕X_{V}}$	mmol / mmol	1.00000
*Km*_,__*thr*_	mmol/L	0.321460	$Y_{Ser∕X_{V}}$	mmol / mmol	947.648
*Km*_,__*trp*_	mmol/L	34.1647	$Y_{Thr∕X_{V}}$	mmol / mmol	64,235.5
*Km*_,__*tyr*_	mmol/L	0.192421	$Y_{Trp∕X_{V}}$	mmol / mmol	4081.34
*Km*_,__*val*_	mmol/L	3.90144	$Y_{Tyr∕X_{V}}$	mmol / mmol	131213
*K*_*S*,__*asn*_	mmol/L	1.81423	$Y_{Val∕X_{V}}$	mmol / mmol	21,899.4
*u*_*max*_	*h*^−1^	0.0443173			

**Table 4:** CCDYN notation.

Symbol	Description
*f*_*c*_	Fraction of cycling cells
*f*_*r*_	Fraction of resting cells
*k*_*d*_	Specific death rate
$k_{d}^{max}$	Maximum Specific death rate
*k*_*l*_	Specific lysis rate
*k*_*N*_	Transport rate constant for the nutrient N
$k_{N}^{p}$	Transport rate constant for the nutrient N in the phase p
*K*_*S*,*M*_	Saturation constant of the metabolite M acting as a substrate
*K*_*I*,*M*_	Saturation constant of the metabolite M acting as an inhibitor
*K*_*m*,*N*_	Saturation constant for the nutrient N in the transport rate equation
$K_{m,N}^{p}$	Saturation constant for the nutrient N in the phase p
*n*_*i*_	Exponent in the Moser’s kinetics
Osm	Osmolality of the cell medium
*Osm*^*b*^	Background osmolality
*Osm*^*iso*^	Isotonic osmolality
*q*_*N*_	Uptake rate of the nutrient N
$q_{N}^{max}$	Maximum uptake rate of the nutrient N
$q_{N}^{max,p}$	Maximum uptake rate of the nutrient N in the phase p
*Q*_*in*_	Volume inlet rate
*Q*_*out*_	Volume outlet rate
t	Time
*V*_*R*_	Volume of cell medium in the bioreactor
*X*_*D*_	Dead cell concentration
*X*_*L*_	Lysed cell concentration
*X*_*V*_	Viable cell concentration
$Y_{M∕X_{V}}$	Specific production/consumption rate of the metabolite M
$Y_{M∕X_{V}^{P}}$	Specific production/consumption rate of the metabolite M in the phase p
*Y*_*M*∕*μ*_	Specific growth-associate yield rate of the metabolite M
*Y*_*M*∕*N*_	Specific nutrient-associate yield rate of the metabolite M
[*M*]	Concentration of the metabolite M
[*M*^*cr*^]	Critical concentration of the metabolite M acting as an inhibitor
[*M*^*E*^]	Excess concentration of the metabolite M
[*N*]	Concentration of the nutrient N consumed by cells
[*N*_*feed*_]	Concentration of the nutrient N in the feeding solution
[*N*_*int*_]	Concentration of the nutrient N in the intracellular environment
[*P*]	Concentration of the product P secreted by cells
[*P*_*feed*_]	Concentration of the product P in the feeding solution
*μ*_*g*_	Gross specific growth rate
$\mu_{g}^{max}$	Maximum gross specific growth rate
$\mu_{g}^{max,iso}$	Maximum gross specific growth rate at isotonic conditions
*μ*_*net*_	Net specific growth rate

## Appendix A Digital twin model of the reactor

### A.1 Cell culture dynamics (CCDyn)

The CCDyn model consists of material balances of the main components (i.e. cells, nutrients and metabolites) and the corresponding specific rates of uptake and production. The volume of the cell culture, *V*_*R*_, varies according to both the sampling and feeding schedule:

(3) $$\frac{dV_{R}}{dt}=Q_{in}-Q_{out}$$

And the material balance for viable cells takes the following format:

(4) $$\frac{d\left(X_{V}V_{R}\right)}{dt}=\mu_{net}X_{V}V_{R}-Q_{out}X_{V}$$

where *X*_*V*_ is the concentration of viable cells (i.e. proliferating and quiescent cells, cells *L*^−1^), and the net specific growth rate is the difference between the gross specific growth rate and the specific death rate (*μ*_*net*_ = *μ*_*g*_ − *kd*).

In this model, it is assumed that, during the exponential phase, all cells are proliferating (cycling cells) at a maximum growth rate $\mu_{g}^{max}$ that can be estimated from the population doubling time. During the decline phase, it is assumed that a fraction of the cells ($X_{V}^{c}$, cycling cells) are still growing at the same maximum growth rate, $\mu_{g}^{max}$, while the remaining fraction are entering G0 ($X_{R}^{c}$, resting cells) and their growth rate is zero. As a consequence, the apparent specific growth rate is defined as

(5) $$\mu_{g}=\mu_{g}^{max}f_{c}+0\cdot f_{r}$$

where the fractions of cycling and resting cells are, respectively:

(6) $$f_{c}=X_{V}^{c}∕X_{V}=\mu_{g}∕\mu_{g}^{max}$$

(7) $$f_{r}=\left(X_{V}^{r}\right)∕X_{V}=\left(X_{V}-X_{V}^{c}\right)∕X_{V}=\mu_{g}^{max}-\mu_{g}∕\left(\mu_{g}^{max}\right)$$

In the stationary phase, all cells are supposed to be quiescent and can perish at the rate of death *k*_*d*_ [42]. This approach allows segregation among proliferating, resting, and dead cell subpopulations to be incorporated into the Monod-like kinetic model, usually unsegregated. Eq. (4) becomes the following:

(8) $$\frac{d\left(X_{V}V_{R}\right)}{dt}=\mu_{g}^{max}\left(f_{c}X_{V}\right)V_{R}-k_{d}\left(f_{r}X_{V}\right)V_{R}Q_{out}X_{V}$$

The dead cell concentration is defined as follows:

(9) $$\frac{d\left(X_{F}V_{R}\right)}{dt}=k_{d}\left(f_{r}X_{V}\right)V_{R}-k_{l}X_{D}V_{R}-Q_{out}X_{V}$$

where *k*_*l*_ is the rate of cell lysis. The amount of biomass that is not accounted for in the total cell number because of lysis can be calculated based on the concentration of lysed cells, *X*_*L*_:

(10) $$\frac{d\left(X_{L}V_{R}\right)}{dt}=k_{l}X_{D}V_{R}-Q_{out}X_{V}$$

The fraction of cycling cells depends on the concentration of growth-limiting nutrients and/or inhibitory metabolites. For the specific case of the cell line under investigation herein and in the range of the medium and the feeding strategies adopted in the experimental campaign [34], the only limiting nutrient is asparagine. Depleting asparagine has been found to cause the decline of cell growth, i.e., arguably, the transition of viable cells from cell division to cell cycle arrest. Cell growth decline has also been experimentally observed when asparagine is still available. For this reason, the inhibitory effect of both ammonium and lactate is considered.

According to the experimental evidence reported [34], the fraction of cycling cells is defined as:

(11) $$f_{c}=1-f_{r}=\frac{\mu_{g}}{\mu_{g}^{max}}=\left(\frac{Asn}{K_{S,Asn}+Asn}\right)\left(\frac{K_{I,Amm}}{K_{I,Amm}+Amm^{E}}\right)$$

where *Amm*^*E*^ is the concentration of ammonium that contributes to cell growth inhibition:

(12) $$Amm^{E}=\begin{array}{c} 0ifAmm\leq Amm^{cr}\\ Amm-Amm^{cr}ifAmm>Amm^{cr} \end{array}$$

*Amm*^*cr*^ is an extracellular concentration threshold, below which it is assumed that there is no adverse effect on cell growth. It is an adjustable model parameter and was found to be equal to 3.9 mM. Since the cell culture experiments were carried out at different osmolality conditions, the maximum cell growth rate is defined as a linear function of culture osmolality [43] to describe the doubling time increase in the exponential growth observed when augmenting osmolality:

(13) $$\mu_{g}^{max}=\mu_{g}^{max,iso}-m\left(Osm-Osm^{iso}\right)$$

$\mu_{g}^{max,iso}$ is the maximum growth rate at isotonic conditions *Osm*^*iso*^ = 290*mOsm*∕*kg*, while the slope *m* = 10^−4^*mOsm*^−1^*h* − 1 is a parameter estimated from experimental data available in the literature [43]. Osmolality is calculated based on the molar concentrations of all the nutrients and metabolic products considered in the model, and the background osmolality (*Osm*^*b*^ = 212*mOsm*∕*kg*) is assumed to be constant with time [44].

(14) $$Osm=Osm^{b}+\overset{N}{\underset{i}{\sum }}M_{i}$$

The cell death has been assumed only to depend on the concentration of ammonia. The first expression that has been adopted is adapted from the literature [45]:

(15) $$k_{d}=k_{d}^{max}{\left(\frac{Amm}{K_{d,Amm}+Amm}\right)}^{2}$$

The concentration profile of the i-th nutrient *N*_*i*_ in the medium varies on time according to the following molar balance:

(16) $$\frac{d\left(N_{i}V_{R}\right)}{dt}=Q_{in}N_{i,feed}-Q_{out}N_{i}-q_{Ni}X_{V}V_{R}$$

while the temporal profile for the i-th catabolite or product *P*_*i*_ is described through the following balance equation:

(17) $$\frac{d\left(P_{i}V_{R}\right)}{dt}=Q_{in}P_{i,feed}-Q_{out}P_{i}-q_{Pi}X_{V}V_{R}$$

*q*_*Ni*_ and *q*_*Pi*_ are the specific uptake and production rates, respectively. In the case of facilitated diffusion (energy-independent), the transport rate equation is analogous to Michaelis-Menten kinetics because substrate binding to the membrane transporters follows saturation kinetics. The most general equation that can be used to describe a saturation reaction is the Moser equation:

(18) $$q_{Ni}=q_{Ni}^{max}\left(\frac{N_{i}^{n_{i}}}{K_{m,Ni}}+N_{i}^{n_{i}}-\frac{N_{i,int}^{n_{i}}}{K_{m,Ni}}+N_{i,int}^{n_{i}}\right)$$

where *N*_*i*,__*int*_ is the intracellular concentration, $q_{Ni}^{max}$, is the maximum uptake rate and $K_{m,N_{i}}$ is the saturation constant which depends on the affinity of the substrate to the membrane. The exponential *n*_*i*_ is an adjustable parameter and assumed equal to 1 for all the nutrient profiles (except for pyruvate, see table of parameters in Appendix A.4). Moreover, the second term on the right-hand side of the Eq. (18) can be neglected under the assumption of fast intracellular consumption.

(19) $$q_{Ni}=q_{Ni}^{max}\left(\frac{N_{i}^{n_{i}}}{K_{m,Ni}}+N_{i}^{n_{i}}\right)$$

In this work, cells will take nutrients from the environment at a specific uptake rate depending on the growth phase. This model assumption is based on the fact that cells enter different phases under different ratios among nutrient (or inhibitor) concentrations, which can lead to a different cellular metabolic state and the activation of different parallel transport systems. Therefore, the specific consumption rate for viable cells is defined such that the segregation between cycling and resting cells is taken into account:

(20) $$q_{Ni}=f_{c}q_{Ni}^{max,c}\left(\frac{N_{i}^{n_{i}}}{K_{m,Ni}}+N_{i}^{n_{i}}\right)+f_{r}q_{Ni}^{max,c}\frac{N_{i,int}^{n_{i}}}{K_{m,Ni}}+N_{i,int}^{n_{i}}$$

The specific uptake rate in both the exponential and stationary phases is expressed through a Michaelis-Menten equation but may differ for cycling and resting cells. These parameters are assumed constant at varied medium concentrations (at least in a given interval).

At relatively low concentrations, the transport rate shows a linear dependence on the concentration:

(21) $$q_{Ni}≃\frac{q_{Ni}^{max}}{K_{m,N_{i}}}N_{i}=k_{N_{i}}N_{i}$$

where $k_{N_{i}}$ is the transport rate constant. This is the case for most of the nutrient profiles considered in this work, whose specific consumption rate can be described with only one parameter per cell category: $k_{N_{i}}^{c}$ for cycling cells or $k_{N_{i}}^{r}$ for resting cells. Some amino acids can be carried through the membrane by two or more transporter systems [46]. However, the fact that transport rates are proportional to the extracellular concentration of most amino acids means that the latter is far from the transporter saturation level, and transport competition can be neglected. Indeed, transport can be described as passive diffusion. At relatively high concentrations, the transport rate is almost constant and equal to the maximum specific rate:

(22) $$q_{Ni}≃q_{Ni}^{max}=Y_{N_{i}∕X_{V}}$$

In the system under investigation, this is the case of the glycine transport rate. Interestingly, unlike other amino acids, glycine is transported bi-directionally through a dedicated transporter slc6a9 [46]. This is also the case for the recombinant protein production rate, which is described as follows:

(23) $$q_{Prod}=f_{c}Y_{Prod∕X_{V}}^{c}+f_{r}Y_{Prod∕X_{V}}^{r}$$

where $Y_{Prod∕X_{V}}^{c}$ is the specific production rate for cycling cells, while $Y_{Prod∕X_{V}}^{r}$ is the specific production rate for resting cells. The production rate also depends on culture osmolality. In general, antibody productivity rate increases with osmolality. Ho and coworkers reported that the productivity rate in GS-NS0 increases by 50% when increasing osmolality from 290 to 450 mOsm/Kg [47]. Unfortunately, a quantitative description of the effect of the medium osmolality on specific antibody productivity is not available for the system under investigation. For this reason, the specific production rates are assumed constant in this work.

The general Eq. (20) cannot be used to describe the concentration profiles of all metabolites that are produced/secreted by cells. In principle, Eq. (18) should be adopted to describe the transport rate, but this would require experimental information about the intracellular concentrations. In the absence of this experimental information, the transport rates for these metabolites are defined as functions of the extracellular environment by inferring the relevant dependencies from metabolic information. This approach has been used to define the transport rate equations for alanine, continually produced by cells, and ammonium, aspartate, glutamine and lactate, consumed and produced by GS-CHO cells. In the cases of these later metabolites, a production term is considered along with the consumption term in the transport rate equation:

(24) $$q_{Mi}=q_{Mi}^{cons}-q_{Mi}^{prod}$$

The consumption term has the general expression reported in Eq. (20), whilst the production term definition depends on the phase. In particular, for cycling cells, the production is assumed to be growth-associate, and a yield coefficient is introduced:

(25) $$q_{Mi}^{prod,c}=\mu_{g}^{max}Y_{Mi∕\mu }$$

For resting cells, the production rate is nutrient-associated, meaning it is assumed to be proportional to the consumption of other specified nutrients after the deduction of what is used for the recombinant protein synthesis:

(26) $$q_{Mi}^{prod,r}=\underset{j}{\sum }q_{Nj}^{r}Y_{Mi∕Nj}-q_{prod}^{r}Y_{Mi∕prod}$$

where *Y*_*Mi*__∕__*prod*_ is the fraction of the i-th metabolite in the recombinant protein composition. The transport rate of metabolites secreted by cells is generally defined by (24), (25), (26).

### A.2 Model parameter estimation and fitting procedure

The cell culture dynamics model was implemented in gPROMS v.4.0.0 (Process Systems Enterprise Ltd) for the experimental batch and all fed-batch conditions. Experimental data and model simulation results were compared through the gPROMS’ Parameter Estimations tool to fit the adjustable model parameters. One single set of parameter values was obtained for the three used datasets. All the obtained parameter values for the metabolites and product are reported in Table 3 (in Appendix A.4).
